# Robot-assisted versus laparoscopic distal pancreatectomy: a systematic review and meta-analysis including patient subgroups

**DOI:** 10.1007/s00464-023-09894-y

**Published:** 2023-02-13

**Authors:** Tess M. E. van Ramshorst, Eduard A. van Bodegraven, Pietro Zampedri, Meidai Kasai, Marc G. Besselink, Mohammad Abu Hilal

**Affiliations:** 1 Department of General Surgery, Istituto Ospedaliero Fondazione Poliambulanza, Brescia, Italy; 2 Department of Surgery, Amsterdam UMC, University of Amsterdam, Amsterdam, The Netherlands; 3 Cancer Center Amsterdam, Amsterdam, The Netherlands; 4 Department of Surgery, Meiwa Hospital, Hyogo, Japan

**Keywords:** Pancreas, Robot-assisted, Laparoscopy, Distal pancreatectomy, Meta-analysis, Subgroups

## Abstract

**Background:**

Robot-assisted distal pancreatectomy (RDP) has been suggested to hold some benefits over laparoscopic distal pancreatectomy (LDP) but consensus and data on specific subgroups are lacking. This systematic review and meta-analysis reports the surgical and oncological outcome and costs between RDP and LDP including subgroups with intended spleen preservation and pancreatic ductal adenocarcinoma (PDAC).

**Methods:**

Studies comparing RDP and LDP were included from PubMed, Cochrane Central Register, and Embase (inception-July 2022). Primary outcomes were conversion and unplanned splenectomy. Secondary outcomes were R0 resection, lymph node yield, major morbidity, operative time, intraoperative blood loss, in-hospital mortality, operative costs, total costs and hospital stay.

**Results:**

Overall, 43 studies with 6757 patients were included, 2514 after RDP and 4243 after LDP. RDP was associated with a longer operative time (MD = 18.21, 95% CI 2.18–34.24), less blood loss (MD = 54.50, 95% CI ﻿− 84.49–24.50), and a lower conversion rate (OR = 0.44, 95% CI﻿ 0.36–0.55) compared to LDP. In spleen-preserving procedures, RDP was associated with more Kimura procedures (OR = 2.23, 95% CI﻿ 1.37–3.64) and a lower rate of unplanned splenectomies (OR = 0.32, 95% CI﻿ 0.24–0.42). In patients with PDAC, RDP was associated with a higher lymph node yield (MD = 3.95, 95% CI ﻿1.67–6.23), but showed no difference in the rate of R0 resection (OR = 0.96, 95% CI﻿ 0.67–1.37). RDP was associated with higher total (MD = 3009.31, 95% CI ﻿1776.37–4242.24) and operative costs (MD = 3390.40, 95% CI ﻿1981.79–4799.00).

**Conclusions:**

RDP was associated with a lower conversion rate, a higher spleen preservation rate and, in patients with PDAC, a higher lymph node yield and similar R0 resection rate, as compared to LDP. The potential benefits of RDP need to be weighed against the higher total and operative costs in future randomized trials.

**Supplementary Information:**

The online version contains supplementary material available at 10.1007/s00464-023-09894-y.

Distal pancreatectomy is the standard treatment for tumors in the body and tail of the pancreas. In recent years, robot-assisted distal pancreatectomy (RDP) and laparoscopic distal pancreatectomy (LDP) have increasingly been adopted. Many studies have suggested the safety, oncologic efficacy, and cost-effectiveness of both techniques as compared to the conventional open distal pancreatectomy (ODP) [1–3]. Two randomized trials have confirmed the superiority of LDP as compared to ODP in terms of time to functional recovery, hospital stay, and intraoperative blood loss [4, 5]. Therefore, the Miami Guidelines on minimally invasive pancreatic resection recommend the use of minimally invasive distal pancreatectomy (MIPD) over ODP for benign and low-grade malignant tumors [6]. For patients with left-sided pancreatic ductal adenocarcinoma (PDAC), guidelines state that in experienced hands minimally invasive distal pancreatectomy appears to be feasible, safe and oncologically equivalent to ODP, although prospective comparative studies are lacking [6].

More recently, interest has shifted towards the comparison between RDP and LDP. Some studies have suggested that RDP is associated with lower conversion rates, lower intraoperative blood loss, higher spleen preservation rates, and reduced hospital stay [7, 8]. On the other hand, RDP carries significantly higher costs which is considered a major drawback [9, 10]. Due to the absence of randomized trials, no superiority of any approach can be claimed. As RDP is associated with high costs, the choice for a robotic approach could include specific patient subgroups who benefit the most from such an approach. Most studies include patients operated for all indications and could, therefore, not advise the surgeon on the preferred approach in a certain patient. Therefore, the choice for RDP or LDP in an individual patient is currently based on the discretion of the operating surgeon, surgeons’ experience, and the availability of the robotic platform, and not on a high level of evidence. To enable future recommendation on the choice for RDP and LDP, more data on outcomes in specific patients subgroups who will benefit from a particular approach is needed.

This systematic review and meta-analysis aims to compare the surgical and oncological outcome of RDP and LDP in unselected patients, patients with intended spleen preservation and patients with PDAC by analyzing the largest number of published studies to date. In addition, a cost-analysis was performed to elaborate on the economic value of both approaches.

## Methods

### Study selection

A systematic review and meta-analysis was performed comparing RDP with LDP. An electronic search was performed in PubMed, Cochrane Central Register of Controlled Trials, and Embase, between inception and July 2022. Search terms included ‘distal pancreatectomy’, ‘minimally-invasive, ‘robot-assisted’ and ‘laparoscopic’ and synonyms. All identified publications were reviewed for inclusion by three reviewers (TVR, EAVB, and PZ) and inconsistencies were addressed by discussion and consensus among the reviewers. The screening process was done according to the PRISMA guidelines [11]. The identified articles were crosschecked on references. The study protocol was registered with PROSPERO (number CRD42022314724).

### Eligibility criteria

Studies comparing RDP versus LDP for all indications and for subgroups were included. Studies with less than 10 patients were excluded. When multiple studies were reported from the same dataset, only the most recent publication was included in the analysis. Letters, editorials, case reports, expert opinions, systematic reviews, and meta-analyses were excluded.

### Outcomes

Primary outcomes were conversion and unplanned splenectomy. Secondary outcomes were R0 resection, lymph node yield, major morbidity, operative time, intraoperative blood loss, in-hospital mortality, operative costs, total costs and hospital stay. Conversion was defined as any procedure that started as a robot-assisted of laparoscopic or procedure but required conversion to open surgery for a reason other than specimen extraction [12]. An unplanned splenectomy was defined as splenectomy in patients operated with the intention to preserve the spleen. Major morbidity was defined as a Clavien-Dindo grade 3a or higher complication [13]. Definition of clinically relevant pancreatic fistula followed the definitions of the International Study Group on Pancreatic Surgery (ISGPS), grade B/C[14] and the type of spleen-preserving procedure was classified according to the Kimura[15] or Warshaw[16] procedure.

### Data extraction and management

A standardized data extraction form was used by the three independent reviewers (TVR, EAVB, and PZ). The following data were extracted from the included studies: first author, year of publication, study design, sample size of the groups, baseline characteristics, surgical details, all primary and secondary outcomes, postoperative care, operative costs and total costs.

### Assessment of risk of bias in included studies

Quality of the studies (all non-RCTs) were assessed using the Newcastle-Ottawa scale [17]. The independent outcomes were assessed with the GRADE approach. Inconsistencies were assessed with the heterogeneity factor p and I^2^. Imprecision was calculated with the Optimal Information Size. Funnel plots were drawn for each outcome and assessed for symmetry to assess publication bias.

### Statistical analysis

A meta-analysis was performed using R (The R Foundation for Statistical Computing, Vienna, Austria, version 4.1.3) with “metafor” and “varameta” package [18]. The results of continuous data (operation time, intraoperative blood loss, lymph node yield, operation cost and hospital stay) were calculated as the mean difference (MD) with 95% confidence intervals (CI’s). For studies reporting only median with range, median and standard deviation were calculated by the “varameta” package. Dichotomous outcome variables were reported as odds ratio’s (OR) with 95% CI’s. Heterogeneity was investigated with the chi-square and I^2^ test and interpreted as follows: 0% to 40% low, 30% to 60% moderate, 50% to 90% substantial, and 75% to 100% considerable. Imprecision of the included studies on the primary outcomes was determined by calculating Optimal Information Size [19]. A fixed effects model was used with a I^2^ index of lower than 50%. A random effects model was used with I^2^ > 50%. A potential publication bias for the primary outcomes was visually inspected by funnel plots and their symmetry was evaluated by Egger’s test [20]. The included studies are displayed in original national currency. Costs were recalculated to 2022 Dutch Euro by using purchasing power parities as provided by the OECD since this study is of Dutch origin. Sensitivity analysis were performed with leave-one-out meta-analysis by excluding each one study at a time to confirm the robustness of our findings [18].

## Results

Overall, 872 studies were identified, of whom 241 duplicates were removed and 548 studies were excluded based on title and abstract. Of the 83 remaining studies, a full text publication could be obtained from 76 studies. Thereafter, 16 studies were excluded because no comparison was made between RDP versus LDP, and 17 further studies were excluded because the required primary outcomes were not reported. No studies were added after a reference crosscheck. Finally, 43 studies were included consisting of six prospective and 37 retrospective studies involving 6757 patients. Of these, 2514 patients underwent RDP and 4243 patients LDP [7, 8, 21–61]. A flowchart of the literature search is shown in Supplementary Fig. 1 and study characteristics in Table 1.

**Table 1:** Characteristics of the included studies

Author	Year	Study period	Study design	Country	*n* RDP/LDP	Age RDP/LDP (as reported)	BMI RDP/LDP (as reported)	Past surgical history RDP/LDP (%)
Alfieri S. [21]	2019	2008–2016	Retrospective	Italy	96/85	NA	NA	48.9/41.1
Baimas-George M. [22]	2020	2009–2019	Retrospective	USA	33/42	68/71^	26.5/25.1^	NA
Beniziri E. [23]	2014	2004–2011	Retrospective	USA	11/23	50.1/52.3*	25.6/26.5*	54.4/43.5
Butturini G. [24]	2015	2011–2014	Prospective	Italy	22/21	54/55^	44.19/25.33^	68.2/61.9
Chen P. [25]	2022	2013–2019	Retrospective	China	54/95	50.06/51.74*	24.23/24.23*	NA
Chen S. [26]	2015	2005–2014	Prospective PSM	China	69/50	56.2/56.5*	24.6/24.6*	0/0
Chopra A. [27]	2021	2008–2019	Retrospective	USA	88/17	NA	NA	65.9/64.7
Daouadi M. [28]	2013	2008–2011	Retrospective	USA	30/94	59/59*	27.9/29*	73/51
De Pastena M. [29]	2020	2011–2017	Retrospective PSM	Italy	37/66	50/53^	24/24^	NA
Di Franco G. [30]	2022	2008–2020	Retrospective PSM	Italy	70/35	Si 60.4 Xi 60.3/63.9^	Si 26.2 Xi 26/26*	NA
Duran H. [31]	2014	2008–2013	Retrospective	Spain	16/18	61/58.3*	NA	NA
Eckhardt S. [32]	2016	2009–2015	Retrospective	Germany	12/29	48.5/59^	23/26.99^	0/0
Esposito A. [33]	2022	1999–2018	Retrospective	Italy	101/300	NA	NA	26.7/20.3
Fisher A.V. [34]	2019	2012–2014	Retrospective	USA	53/146	59/58^	NA	NA
Goh B. K. P. [35]	2017	2006–2015	Retrospective	Singapore	8/31	57/56^	27.6/23.9^	12.5/32.3
Han J. H. [36]	2018	2012–2018	Retrospective	South Korea	13/22	46.1/58.3*	20.9/23.9*	30.8/22.7
Hong S. [37]	2020	2015–2017	Retrospective	South Korea	46/182	51.2/60.2*	24.9/24.6*	32.6/28
Ito M. [38]	2014	2009–2013	Retrospective	Japan	4/10	52.7/68*	NA	NA
Jiang Y. [39]	2020	2011–2018	Retrospective	China	63/103	44.5/48.8*	22.8/22.6*	NA
Kamarajah S. [40]	2022	2007–2018	Retrospective	UK	40/47	62/67^	28/28^	NA
Kang C. [41]	2010	2006–2010	Retrospective	South Korea	20/25	44.5/56.5*	24.2/23.4*	NA
Kriger A.G. [42]	2015	2009–2014	Retrospective	Russia	19/10	49.88/47.4*	NA	NA
Kwon J. [8]	2021	2015–2020	Retrospective PSM	South Korea	104/208	50.62/51.23*	24.05/24.06*	NA
Lai E. C. [43]	2015	1999–2015	Retrospective	China	17/18	61.2/63.2*	24.1/25.7*	NA
Lee S. Q. [44]	2020	2006–2019	Retrospective	Singapore	27/75	64/61^	23.1/23.4^	18.5/30.7
Lee S. Y. [45]	2015	2000–2013	Retrospective	USA	37/131	58/58*	28.7/28.2*	NA
Lin X.C. [46]	2019	2016–2018	Retrospective PSM	China	41/41	45.2/47.4*	NA	NA
Liu R. [47]	2017	2011–2015	Retrospective PSM	China	102/102	48.1/49.62*	NA	NA
Lof S. [7]	2021	2011–2019	Retrospective PSM	NL	402/402	57/57*	25.4/25.9*	41/38.3
Lyman W.B. [48]	2019	2008–2017	Retrospective	USA	108/139	56.3/59.5*	29.3/29*	NA
Magge D. [49]	2018	2010–2016	Retrospective	USA	196/93	62.7/61.3*	29.68/28.21*	NA
Marino M. [50]	2020	2014–2017	Retrospective PSM	Italy	35/35	59.3/58.5^	NA	20/14.3
Najafi N. [51]	2020	2008–2015	Retrospective	Germany	24/32	NA	NA	NA
Qu L. [52]	2018	2011–2015	Retrospective PSM	China	35/35	58.1/57.8*	24.46/24.08*	NA
Raoof M. [53]	2018	2010–2013	Retrospective	USA	99/605	NA	NA	NA
Rodriguez M. [54]	2018	2012–2015	Retrospective	France	21/25	53/62.5^	25/27.3^	71.4/68
Ryan C. E. [55]	2015	2012–2014	Prospective	USA	18/16	68/58*	28/25*	NA
Souche R. [56]	2018	2011–2016	Prospective	France	15/23	57/66^	23/25^	13/21
Vicente E. [57]	2020	2011–2018	Prospective	Spain	31/28	59.9/61.5^	24.2/24.5^	NA
Waters J. A. [58]	2010	2008–2009	Prospective	USA	17/18	64/59"	NA	NA
Xourafas D. [59]	2017	Jan 2014–Dec 2014	Retrospective	USA	200/694	62/62^	28.8/28.4^	NA
Yang S. J. [60]	2020	2007–2018	Retrospective	South Korea	37/41	42.9/51.3*	23.5/24.1*	NA
Zhang J. [61]	2017	2010–2017	Retrospective	China	43/31	47.9/48.7*	23.9/23.3*	NA

*Mean, ^median, “unknown, *PSM* Propensity Score Matching, *RDP* robotic distal pancreatectomy, *LDP* laparoscopic distal pancreatectomy, *BMI* Body Mass Index, *NA* not applicable

### Risk of bias assessment

The risk of bias is displayed in Supplementary Table 1. None of the included studies had a very high risk of bias (0 to 3 points) and the minimum risk of included studies was 7. Inconsistency was determined based on the heterogeneity factor p and I^2^ as shown in Table 2. For the primary outcomes, conversion and unplanned splenectomy, a low heterogeneity was found. For the secondary outcomes R0 resection, major morbidity and in-hospital mortality, a low heterogeneity was found, whereas for operative time, intraoperative blood loss, lymph node yield, operative costs, total costs and hospital stay a substantial heterogeneity was found. With an event rate between both groups of 36.7% for conversions and 54.4% for unplanned splenectomy, the optimal information size threshold (*n* = 2766) was met for the primary outcomes with an overall sample size of 6757 in this study.

**Table 2:** Summary of findings with GRADE

Outcome	No of studies	Risk of bias	Inconsistency	Indirectness	Imprecision	Quality GRADE	Statistical method	Effect estimate
Operative time in minutes	40	NS^a^	*p* = 0.00 I^b^ = 90.5%	NS^b^	NS^c^	Mod ⊕ ⊕ ⊕ ⊝	Mean difference (REM, 95% CI)	18.21 [2.18, 32.24]
Intraoperative blood loss in ml	34	NS^a^	*p* = 0.00 I^b^ = 91.9%	NS^b^	NS^c^	Mod ⊕ ⊕ ⊕ ⊝	Mean difference (REM, 95% CI)	− 54.50 [− 84.49, − 24.50]
Conversion	39	NS^a^	*p* = 0.45 I^b^ = 1.1%	NS^2^	NS^c^	High ⊕ ⊕ ⊕ ⊕	Odds Ratio (M–H, FEM, 95% CI)	0.44 [0.36, 0.55]
Unplanned splenectomy	15	NS^a^	*p* = 0.19 I^b^ = 23.7%	NS^b^	NS^c^	Mod ⊕ ⊕ ⊕ ⊝	Odds Ratio (M–H, REM, 95% CI)	0.32 [0.24, 0.42]
Kimura	20	NS^a^	*p* = 0.02 I^b^ = 53.0%	NS^b^	NS^c^	Mod ⊕ ⊕ ⊕ ⊝	Odds Ratio (M–H, REM, 95% CI)	2.23 [1.37, 3.64]
Blood transfusion	22	NS^a^	*p* = 0.68 I^b^= 0.0%	NS^b^	NS^c^	Mod ⊕ ⊕ ⊕ ⊝	Odds Ratio (M–H, FEM, 95% CI)	0.93 [0.69, 1.25]
Major morbidity	31	NS^a^	*p* = 0.31 I^b^ = 9.7%	NS^b^	NS^c^	Mod ⊕ ⊕ ⊕ ⊝	Odds Ratio (M–H, FEM, 95% CI)	0.93 [0.76, 1.14]
POPF	40	NS^a^	*p* = 0.89 I^b^ = 0.0%	NS^b^	NS^c^	Mod ⊕ ⊕ ⊕ ⊝	Odds Ratio (M–H, FEM, 95% CI)	0.98 [0.85, 1.14]
Reoperation	25	NS^a^	*p* = 0.84 I^b^ = 0.0%	NS^2^	NS^c^	Mod ⊕ ⊕ ⊕ ⊝	Odds Ratio (M–H, FEM, 95% CI)	0.94 [0.68, 1.31]
In-hospital mortality	31	NS^1^	*p* = 1.00 I^b^ = 0.0%	NS^b^	NS^c^	Mod ⊕ ⊕ ⊕ ⊝	Odds Ratio (M–H, FEM, 95% CI)	1.40 [0.70, 2.82]
Hospital stay in days	32	NS^a^	*p* = 0.00 I^2^ = 71.3%	NS^b^	NS^c^	Low ⊕ ⊕ ⊝ ⊝	Mean difference (REM, 95% CI)	− 0.45 [− 0.92, 0.01]
R0 resections in PDAC	11	NS^a^	*p* = 0.46 I^b^ = 0.0%	NS^b^	NS^c^	Mod ⊕ ⊕ ⊕ ⊝	Odds Ratio (M–H, FEM, 95% CI)	0.96 [0.67, 1.37]
Harvested lymph nodes	10	NS^a^	*p* = 0.00 I^b^= 80.2%	NS^b^	NS^c^	Mod ⊕ ⊕ ⊕ ⊝	Mean difference (REM, 95% CI)	3.95 [1.67, 6.23]
Operative costs	7	NS^a^	*p* = 0.00 I^b^ = 99.5%	NS^b^	NS^c^	Mod ⊕ ⊕ ⊕ ⊝	Mean difference (REM, 95% CI)	3390.40 [1981.79, 4799.00]
Total costs	9	NS^a^	*p* = 0.00 I^b^ = 95.0%	NS^b^	NS^c^	Mod ⊕ ⊕ ⊕ ⊝	Mean difference (REM, 95% CI)	3009.31 [1776.37, 4242.25]

*POPF* postoperative pancreatic fistula; *NS* Not serious; *MD* Mean difference; *REM* random effects model; *FEM* fixed effects model

^a^According to the assessment of risk of bias, the included studies all have a low risk of bias (supplementary Table 1)

^b^The results of these variables for the included studies have no serious effect on the indirectness since the studies relate well to the aim of current study

^c^To determine if imprecision was an influence on the quality of the studies, the Optimal Information Size was calculated using the GRADE approach for the outcome of major morbidity. With an event rate between groups of 36.7% for conversions, 54,4% for unplanned splenectomy and 82.3% for Kimura, the optimal information size threshold was met for the primary outcomes since this implicates that a sample size of minimally 2766 is required

### Publication bias

Funnel plots of publications reporting on the outcomes of interest were symmetrical and all statistically verified (Egger’s test; conversion: *p* = 0.35, unplanned splenectomy: *p* = 0.14, major morbidity: *p* = 0.14, in-hospital; mortality: *p* = 0.71, CR-POPF: *p* = 0.35, reoperation: *p* = 0.47, intraoperative blood transfusion: *p* = 0.19, intraoperative blood loss: *p* = 0.71, operative time: *p* = 0.87, hospital stay: *p* = 0.05, R0 resection: *p* = 0.32, lymph node yield: *p* = 0.09, operation costs: *p* = 0.75, total costs: *p* = 0.61). The funnel plots for the primary outcomes are shown in Supplementary Fig. 2a (conversion) and 2b (unplanned splenectomy).

### Total cohort

#### Preoperative characteristics

The meta-analyses of preoperative patient and tumor characteristics are shown in Supplementary Fig. 3a–d. The RDP cohort included younger patients (MD − 1.66 years, 95% CI: − 2.42 to -0.89) with smaller tumors (MD − 2.75 mm, 95% CI: − 4.52 to -0.98) and more patients with previous abdominal surgery (OR: 1.22, 95% CI: 1.01 to 1.48). BMI did not differ between the RDP and LDP group surgery (MD − 0.10 kg/m^2^, 95% CI: − 0.37 to 0.17).

#### Perioperative outcome

**Fig. 1:**
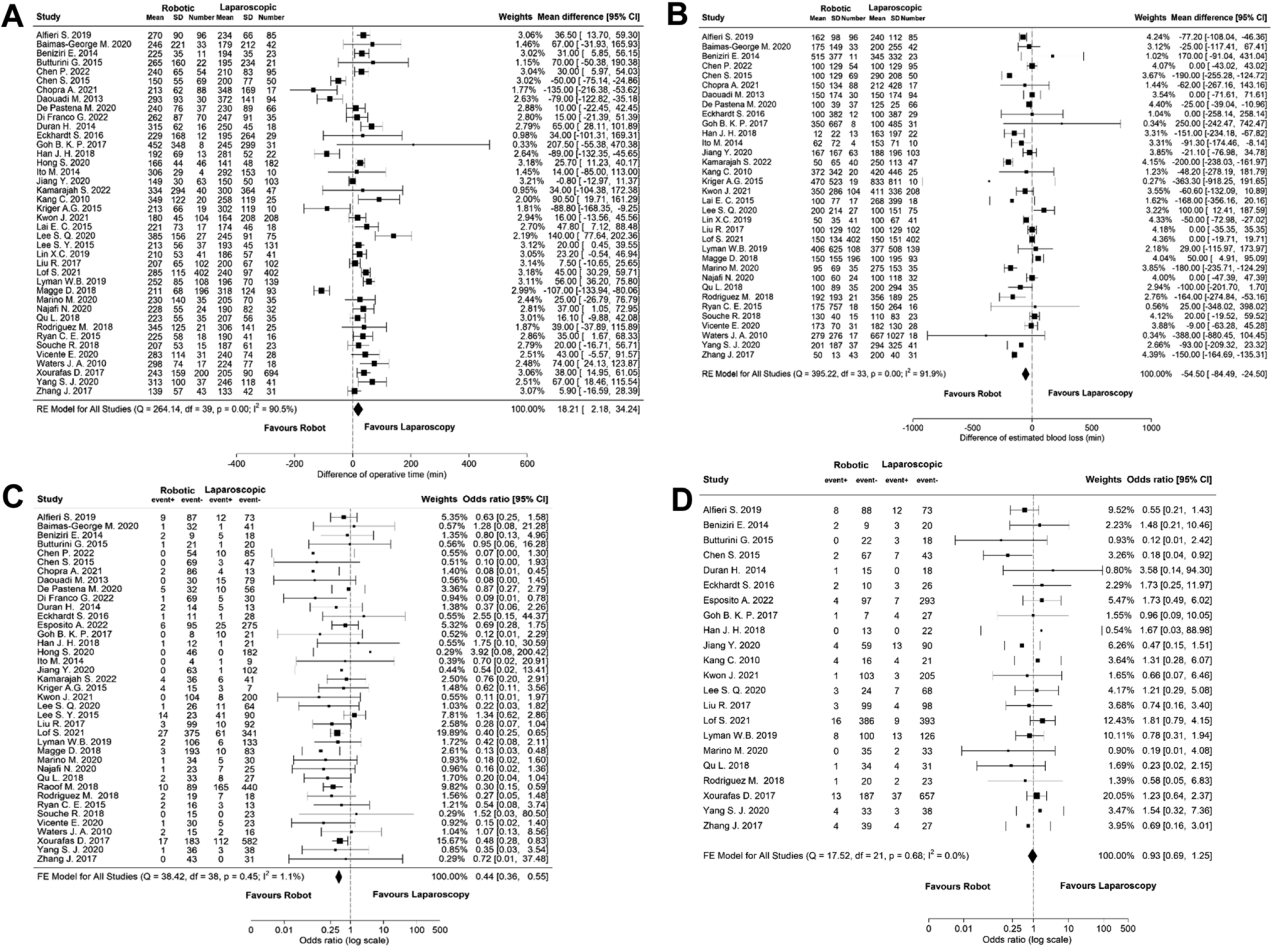
Meta-analyses of the perioperative outcomes of the total cohort; **A** operative time, **B** intraoperative blood loss, **C** conversion, **D** intraoperative blood transfusion

The forest plots of perioperative outcomes are displayed in Fig. 1a–d. RDP was associated with a significantly longer operative time (MD 18.21 min, 95% CI: 2.18 to 34.24) but less intraoperative blood loss (MD − 54.50 mL, 95% CI: − 84.49 to − 24,50) compared to LDP with no significant difference between both groups regarding the rate of intraoperative blood transfusion (OR 0.93, 95% CI: 0.65 to 1.25). The conversion rate was significantly lower in RDP (OR 0.44, 95% CI: 0.36 to 0.55).

#### Postoperative outcome

**Fig. 2:**
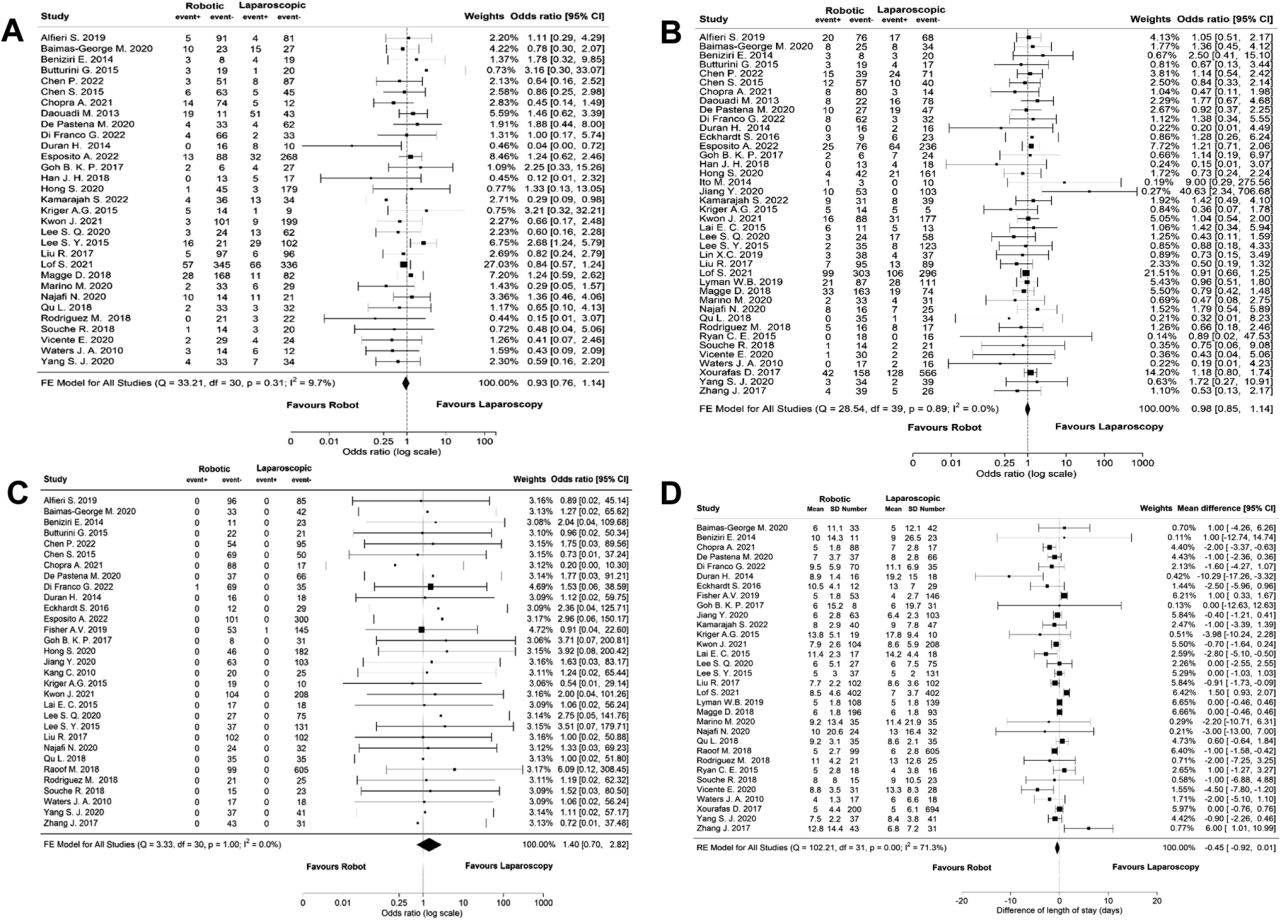
Meta-analyses of the postoperative outcomes of the total cohort; **A** major morbidity, **B** postoperative pancreatic fistula, **C** in-hospital mortality, **D** hospital stay

No significant differences were observed between RDP and LDP regarding all postoperative outcomes. The meta-analyses of major morbidity, POPF, in-hospital mortality and hospital stay are shown in Figs. 2a–d. The shorter hospital stay in the RDP group was not statistically significant (MD − 0.45 days, 95% CI: − 0.92 to 0.01).

### Subgroup analysis splenic preservation

**Fig. 3:**
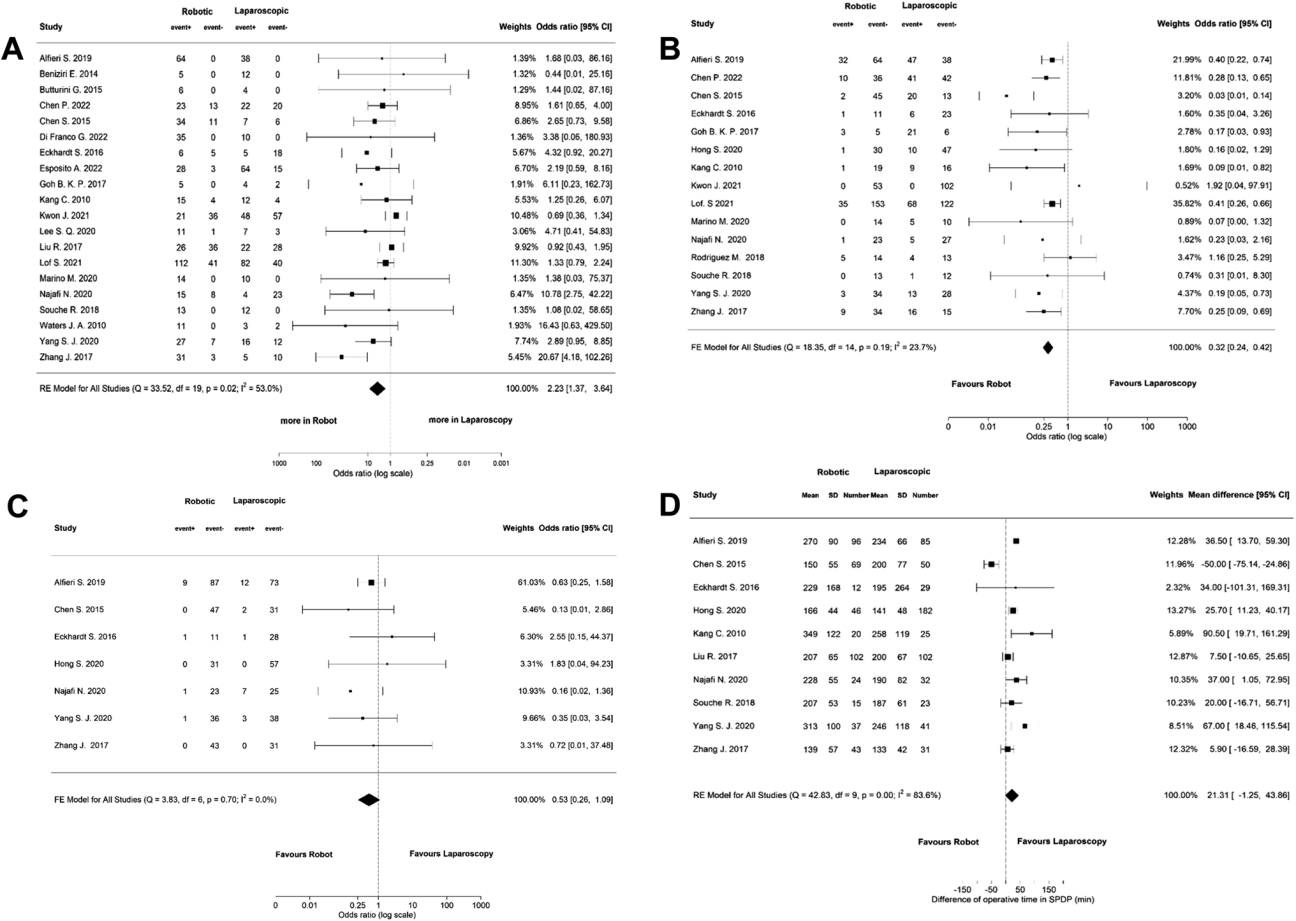
Meta-analyses of the outcomes in patients with intended spleen preservation; **A** Kimura technique, **B** unplanned splenectomy, **C** conversion, **D** operative time

Of the 43 included studies, 20 reported outcomes specifically for spleen-preserving distal pancreatectomy. Meta-analysis of these studies revealed that significantly more Kimura (i.e. splenic vessel preserving) procedures were performed in the RDP group (Fig. 3a, OR 2.23, 95% CI: 1.37 to 3.64). In total, 15 studies assessed the rate of unplanned splenectomy and meta-analysis showed a significantly lower rate of unplanned splenectomies in the RDP group (Fig. 3b, OR 0.32, 95% CI: 0.24 to 0.42). The rate of conversion in these patients did not differ between both groups (Fig. 3c, OR 0.53, 95% CI: 0.26 to 1.09). Operative time was reported in 10 studies, showing no significant difference between RDP and LDP (Fig. 3d, MD 21.31, 95% CI: -1.25 to 43.86).

### Subgroup analysis PDAC

**Fig. 4:**
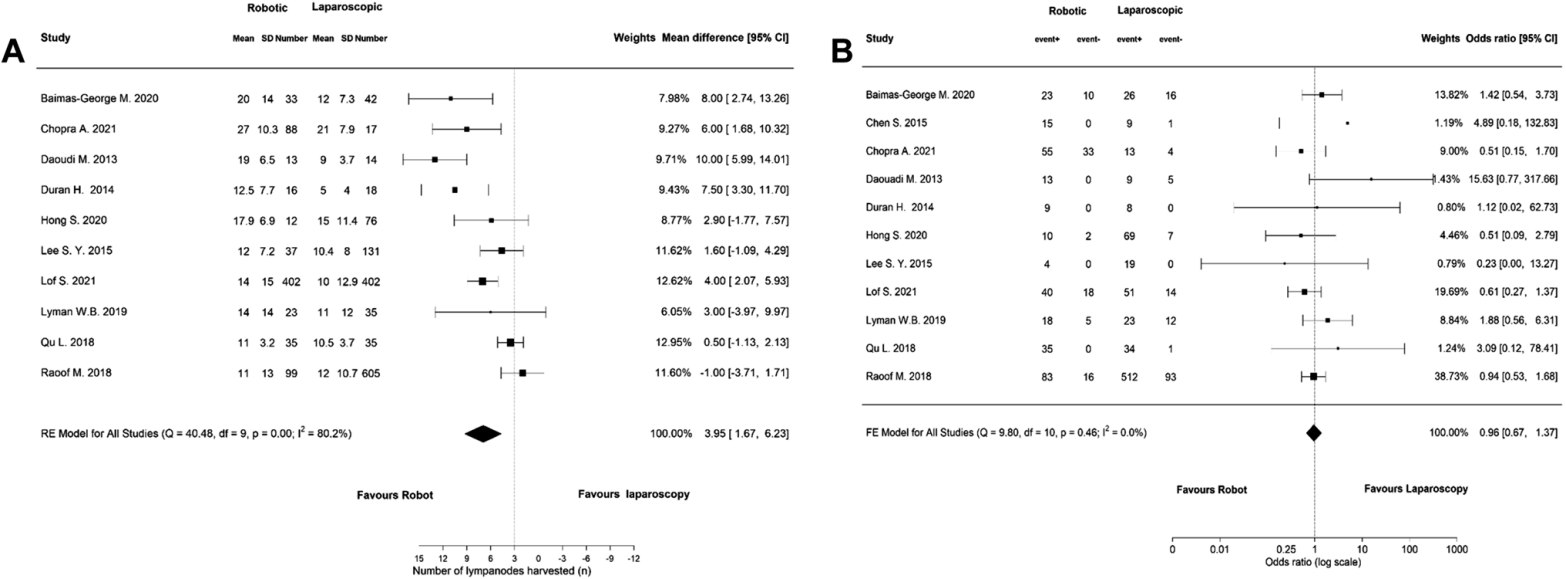
Meta-analyses of the oncological outcomes in patients with PDAC; **A** lymph node yield, **B** R0 resection

Of the 43 included studies, 11 reported on oncological outcomes specifically in patients with PDAC. Meta-analyses of these studies revealed a significant higher lymph node yield in the RDP group compared to LDP (Fig. 4a, MD 3.95 95% CI: 1.67 to 6.23), but no difference in the rate of R0 resection (Fig. 4b, OR 0.96, 95% CI: 0.67 to 1.37). Five studies reported on overall survival and three studies on disease-free survival but the data were insufficient to perform a meta-analysis.

### Cost analysis

**Fig. 5:**
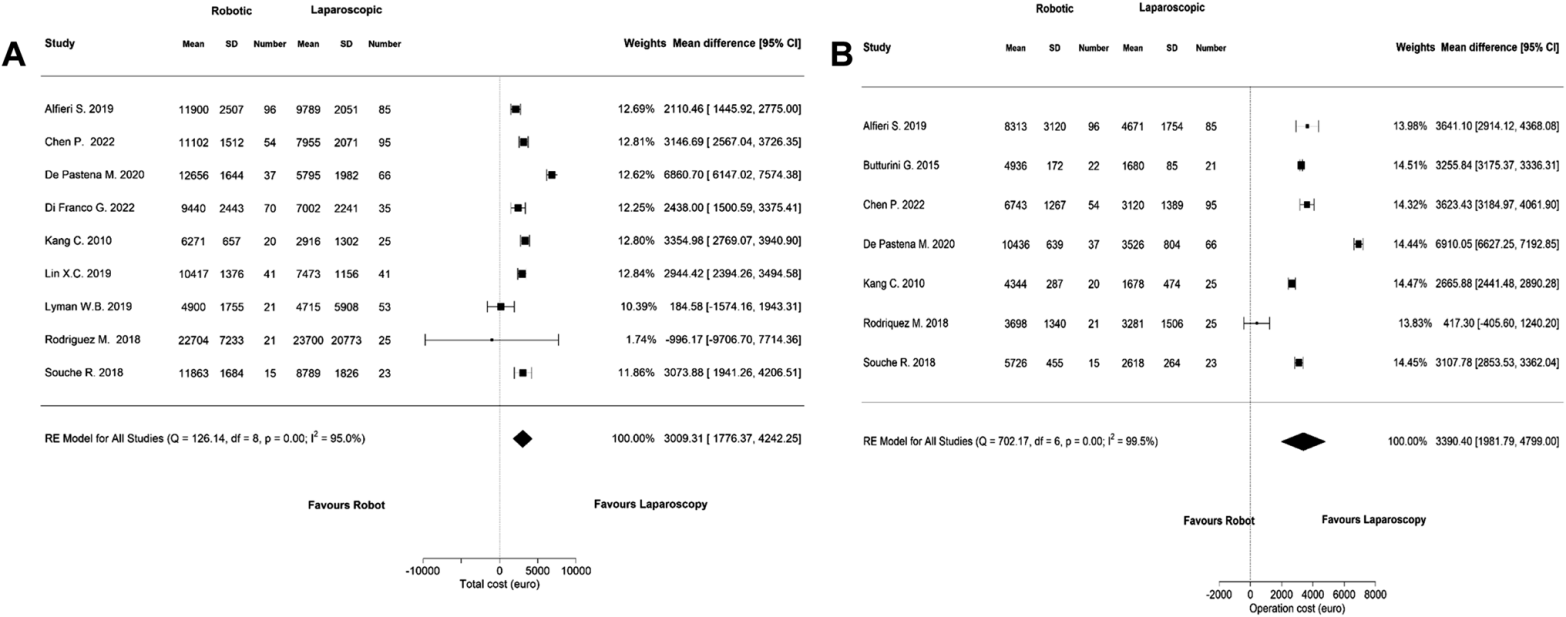
Meta-analyses of the costs; **A** total costs, **B** operative costs

Nine studies reported on the total costs of RDP and LDP and meta-analysis of these studies showed that RDP was significantly more expensive than LDP (Fig. 5a, MD 3009.31, 95% CI: 1776.37 to 4242.24). Operative costs were reported in seven studies and were also significantly higher in RDP (Fig. 5b, MD 3390.40, 95% CI: 1981.79 to 4799.00).

### Leave-one-out analysis

In the leave-one-out analyses, focusing only on the significant differences identified, only previous abdominal surgery showed sensitivity and was no longer significant different between RDP and LDP when leaving out one of the following studies: Alfieri S. 2019, *p* = 0.066 [21], Daouadi M. 2019, *p* = 0.094 [28], Esposito A. 2022, *p* = 0.088 [33].

## Discussion

In this largest systematic review and meta-analysis to date, including specific subgroups, RDP was associated with a lower conversion rate and, in patients with intended spleen preservation, with less unplanned splenectomies and a higher rate of splenic vessels preserving Kimura procedures. RDP was also associated with less intraoperative blood loss as compared to LDP at the cost of longer operative time. In patients with PDAC, RDP showed a higher lymph node yield with comparable R0 rates, as compared to LDP. As expected, RDP was associated with higher costs, as compared to LDP, approximating EUR 3000 per procedure.

In recent years, along with the increasing implementation of minimally invasive distal pancreatectomy, several meta-analyses comparing RDP and LDP have been published [9, 10, 62–70]. However, most of them are obsolete today, reported on half of the available evidence to date or included all indications without distinguishing subgroups. The current systematic review provides a complete and up-to-date analysis, including the most recent studies and around double the number of patients compared to the two most recent meta-analyses on RDP versus LDP for all indications [62, 64], thus contributing to the highest body of evidence in the absence of randomized trials. Moreover, analyses were performed in specific subgroups to demonstrate potential benefits of a particular approach.

Previous meta-analyses also described lower conversion rates of RDP and most of them found longer operative times in RDP [62–64]. Although the outcomes on operative time varied in previous literature, the current study confirmed the prolonged operative time of RDP. This could, at least partially, be explained by the additional time required for preparation and docking of the robot. With respect to other perioperative factors, RDP was associated with less intraoperative blood loss in this study, a finding which has found significant in only two previous meta-analyses potentially because of a type II error [65, 70]. It is assumed that the robotic platform allows for better prevention and control of bleeding due to the greater instrument dexterity, 3D high-definition visualization, and tremor filtration.

In the subgroup group analysis of patients with an intended spleen preservation, RDP was associated with a higher rate of Kimura procedures. A recent meta-analysis of only SPDP studies reported a rate of 81.1% Kimura procedures in the RDP group versus 54.5% in the LDP group, but did not assess its significance in a forest plot [70]. The present study corroborates these findings by showing significance in a forest plot. In general, the Kimura technique is regarded as the preferred procedure in patients planned for a spleen-preserving procedure when there is no tumor proximity or involvement to the splenic vessels [71], which is confirmed by a survey from 2018 that concluded that 82.5% of the surgeons attempt a Kimura procedure if feasible [72]. However, this approach is considered technically challenging due to the difficulty of separating the splenic vessels and dividing their branches from the pancreas. The technical features of the robot may be advantageous in this regard, which could be a reasonable explanation for the higher proportion of Kimura procedures in the RDP group. Interestingly, in SPDP, RDP was no longer associated with a longer operative time as compared to LDP. This may indicate that in such technically complex procedures, RDP loses its relative disadvantage of a longer operative time. In addition, in the subgroup analysis of SPDP, a lower rate of unplanned splenectomies was observed in the RDP cohort compared to the LDP cohort, what aligns with the often described higher spleen preservation rates of RDP in previous meta-analyses [62, 64, 70].

Oncological results of the subgroup of patients with PDAC revealed a higher lymph node yield in RDP with similar R0 resection rates compared to LDP based on 11 included studies. Studies comparing RDP with LDP for PDAC are scarce, but a recently published meta-analysis included six studies that reported outcomes for PDAC [68]. The results of that study showed opposite results to the present study, as RDP was associated with a higher R0 resection rate but a similar lymph node yield compared to LDP. However, only six studies were included for the R0 resection and five studies for the lymph node yield analyses. Contrarily, the current study included almost double that number of studies, with 11 studies on R0 resection rates and 10 studies on lymph node yield.

The results of this study should be interpreted in light of some limitations. First, the current study analyzed several patient and tumor characteristics and found that patients in the RDP group were significantly younger and had smaller tumors. This might indicate that in the first phase of the implementation of RDP more easily operable patients and tumors were selected for a robot-assisted approach. Despite this being an interesting finding, it is also a limitation of the study as it may have contributed to some outcomes, such as the lower blood loss. Second, all of the included studies were observational cohort studies and no randomized controlled trials are yet available. Additional selection bias, other than the identified differences, is therefore likely present, even though studies did attempt to minimize the bias by, for example, correct for confounding through matching of the cohorts. Third, data on 1- and 3-year survival were lacking in the majority of the studies so no firm conclusions can be drawn on survival differences between RDP and LDP. This important oncological outcome has still to be proven by future prospective data. The main strength of this meta-analysis is that it included the largest number of studies and patients to date (43 studies, 6757 patients) as compared to the largest in current literature (21 studies, 3463 patients) [64]. With additional analyses on subgroups and costs, while adopting a robust and more comprehensive method to minimize all potential forms of bias, the current study provides the highest level of evidence on the comparison between RDP and LDP.

## Conclusions

This systematic review and meta-analysis found RDP associated with a higher rate of spleen preservation, a lower conversion rate, and similar postoperative outcomes as compared to LDP. RDP seems to be an oncological safe alternative to LDP given the equal R0 resection rate and higher lymph node yield. Potential disadvantages of RDP are the higher costs and longer operative time. Based on these results, and acknowledging the potential impact of bias in patients selection, RDP may be preferred over LDP in patients with benign lesions planned for a complex or Kimura intended spleen-preserving procedure. However, future randomized controlled trials are needed to confirm these findings and weigh the potential benefits and downsides of RDP with the associated costs.

## Supplementary Information

Below is the link to the electronic supplementary material.

Supplementary file1 (PPTX 37 kb)

Supplementary file2 (PPTX 209 kb)

Supplementary file3 (PPTX 1132 kb)

Supplementary file4 (DOCX 17 kb)
