# Supplementary material for: Robot-assisted versus laparoscopic distal pancreatectomy: a systematic review and meta-analysis including patient subgroups
Source: Surg Endosc. 2023 Feb 13;37(6):4131–43. doi: 10.1007/s00464-023-09894-y (PMC10235152; doi:10.1007/s00464-023-09894-y)
Supplement: Supplementary file 1 — Supplementary file1 (PPTX 37 kb) [file 464_2023_9894_MOESM1_ESM.pptx]

## Slide 1
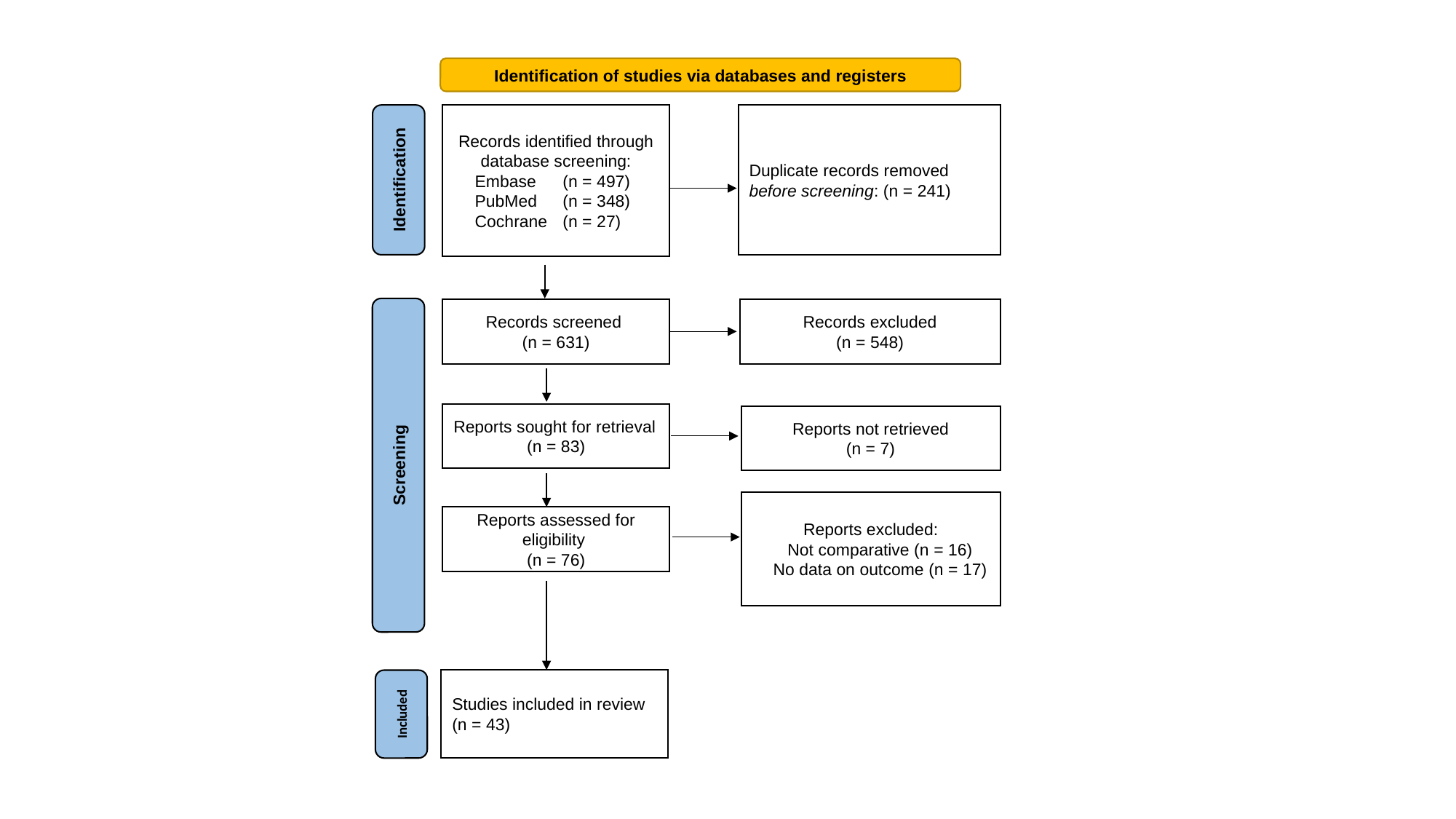

Identification of studies via databases and registers
Identification
Records identified through database screening:
Embase 	(n = 497)
PubMed 	(n = 348)
Cochrane 	(n = 27)
Duplicate records removed
before screening: (n = 241)
Screening
Records screened
(n = 631)
Records excluded
(n = 548)
Reports sought for retrieval
(n = 83)
Reports not retrieved
(n = 7)
Reports excluded:
 Not comparative (n = 16)
 No data on outcome (n = 17)
Reports assessed for eligibility
(n = 76)
Studies included in review
(n = 43)
Included
