# Supplementary figures and images for: Robot-assisted versus laparoscopic distal pancreatectomy: a systematic review and meta-analysis including patient subgroups
Source: Surg Endosc. 2023 Feb 13;37(6):4131–43. doi: 10.1007/s00464-023-09894-y (PMC10235152; doi:10.1007/s00464-023-09894-y)

## Slide 1
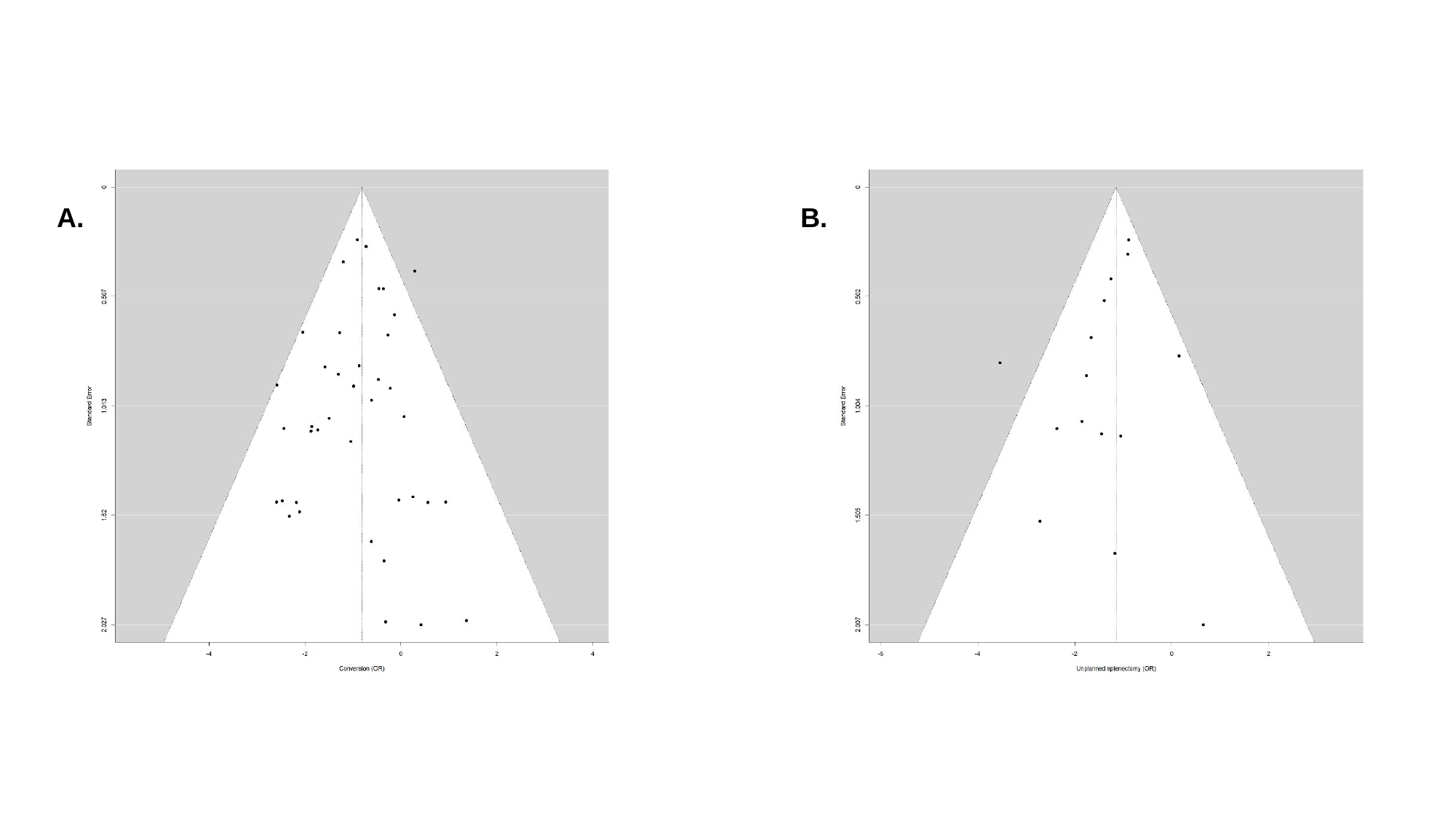

A.
B.

Supplement: Supplementary file 2 — Supplementary file2 (PPTX 209 kb) [file 464_2023_9894_MOESM2_ESM.pptx]

## Slide 1
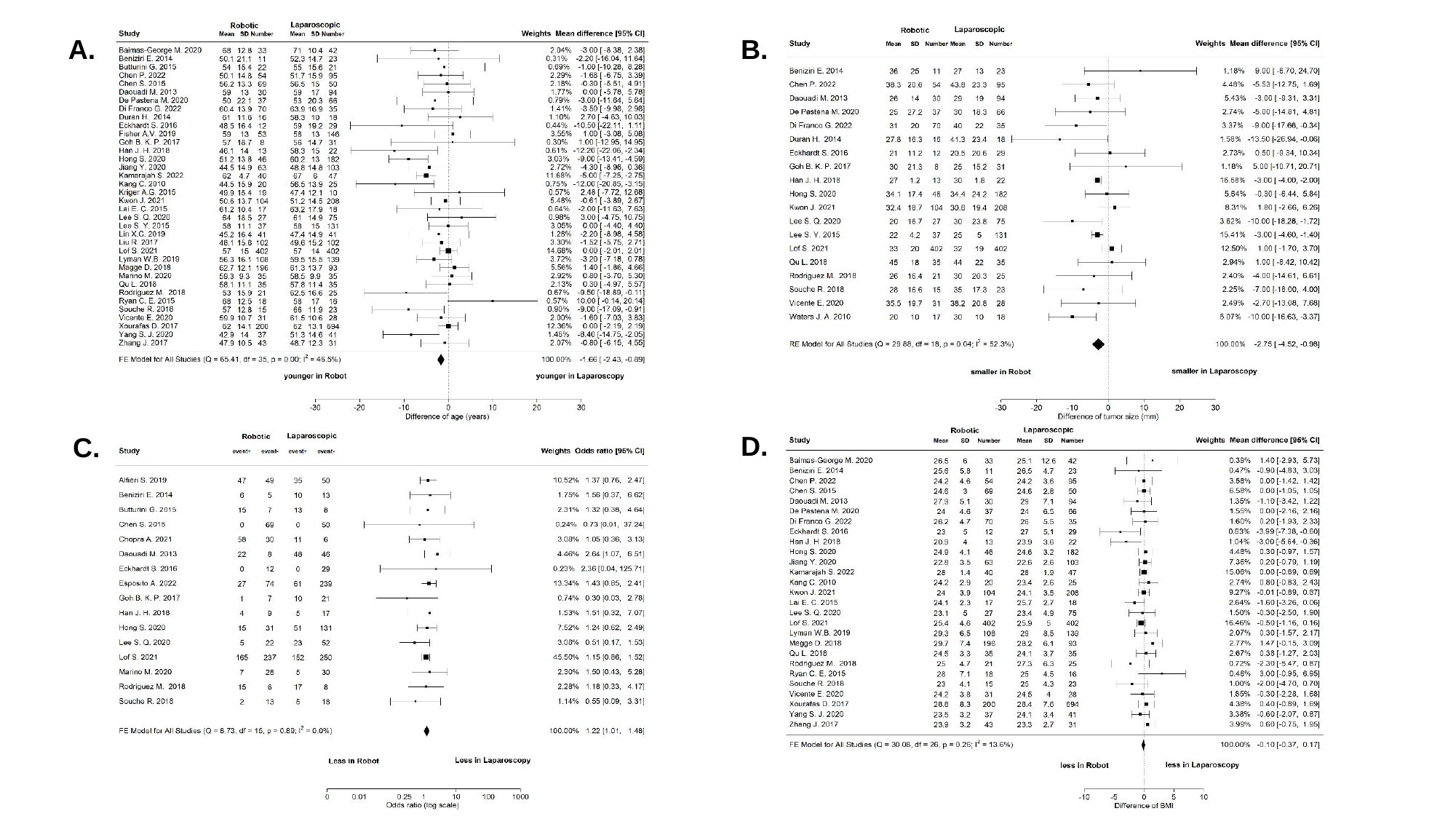

A.
B.
D.
C.

Supplement: Supplementary file 3 — Supplementary file3 (PPTX 1132 kb) [file 464_2023_9894_MOESM3_ESM.pptx]
