# Supplementary material for: Robot-assisted versus laparoscopic distal pancreatectomy: a systematic review and meta-analysis including patient subgroups
Source: Surg Endosc. 2023 Feb 13;37(6):4131–43. doi: 10.1007/s00464-023-09894-y (PMC10235152; doi:10.1007/s00464-023-09894-y)
Supplement: Supplementary file 4 — Supplementary file4 (DOCX 17 kb) [file 464_2023_9894_MOESM4_ESM.docx]

**SUPPLEMENTARY TABLE 1.** Non-RCTs quality assessment using the Newcastle-Ottawa scale.

| **AUTHOR** | **YEAR** | **SELECTION** | | | | **COMPARABILITY** | | **OUTCOME** | | | **SCORE** |
| --- | --- | --- | --- | --- | --- | --- | --- | --- | --- | --- | --- |
|  | | Exposed truly representative of average | Selection of non-exposed from the same community | Exposure ascertained by secure record or interview | Demonstration of outcome of interest not present at the start of the study | Study controls for preoperative comparability | Study controls for operative comparability | Adequate assessment and description of Outcome | Was Follow-Up Long Enough for Outcomes to Occur | Adequacy of Follow Up of Cohorts |  |
| Alfieri S. | 2019 | X | X | X | X |  |  | X | X | X | 7 |
| Baimas-George M. | 2020 | X | X | X | X |  |  | X | X | X | 7 |
| Beniziri E. | 2014 | X | X | X | X |  |  | X | X | X | 7 |
| Butturini G. | 2015 | X | X | X | X |  |  | X | X | X | 7 |
| Chen P. | 2022 | X | X | X | X |  |  | X | X | X | 7 |
| Chen S. | 2015 | X | X | X | X | X |  | X | X | X | 8 |
| Chopra A. | 2021 | X | X | X | X |  |  | X | X | X | 7 |
| Daouadi M. | 2013 | X | X | X | X |  |  | X | X | X | 7 |
| De Pastena M. | 2020 | X | X | X | X | X |  | X | X | X | 8 |
| Di Franco G. | 2022 | X | X | X | X | X |  | X | X | X | 8 |
| Duran H. | 2014 | X | X | X | X |  |  | X | X | X | 7 |
| Eckhardt S. | 2016 | X | X | X | X |  |  | X | X | X | 7 |
| Esposito A. | 2022 | X | X | X | X |  |  | X | X | X | 7 |
| Fisher A.V. | 2019 | X | X | X | X |  |  | X | X | X | 7 |
| Goh B. K. P. | 2017 | X | X | X | X |  |  | X | X | X | 7 |
| Han J. H. | 2018 | X | X | X | X |  |  | X | X | X | 7 |
| Hong S. | 2020 | X | X | X | X |  |  | X | X | X | 7 |
| Ito M. | 2014 | X | X | X | X |  |  | X | X | X | 7 |
| Jiang Y. | 2020 | X | X | X | X |  |  | X | X | X | 7 |
| Kamarajah S. | 2022 | X | X | X | X |  |  | X | X | X | 7 |
| Kang C. | 2010 | X | X | X | X |  |  | X | X | X | 7 |
| Kriger A.G. | 2015 | X | X | X | X |  |  | X | X | X | 7 |
| Kwon J. | 2021 | X | X | X | X | X | X | X | X | X | 9 |
| Lai E. C. | 2015 | X | X | X | X |  |  | X | X | X | 7 |
| Lee S. Q. | 2020 | X | X | X | X |  |  | X | X | X | 7 |
| Lee S. Y. | 2015 | X | X | X | X |  |  | X | X | X | 7 |
| Lin X.C. | 2019 | X | X | X | X | X |  | X | X | X | 8 |
| Liu R. | 2017 | X | X | X | X | X |  | X | X | X | 8 |
| Lof S. | 2021 | X | X | X | X | X |  | X | X | X | 8 |
| Lyman W.B. | 2019 | X | X | X | X |  |  | X | X | X | 7 |
| Magge D. | 2018 | X | X | X | X |  |  | X | X | X | 7 |
| Marino M. | 2020 | X | X | X | X | X |  | X | X | X | 8 |
| Najafi N. | 2020 | X | X | X | X |  |  | X | X | X | 7 |
| Qu L. | 2018 | X | X | X | X | X |  | X | X | X | 8 |
| Raoof M. | 2018 | X | X | X | X |  |  | X | X | X | 7 |
| Rodriguez M. | 2018 | X | X | X | X |  |  | X | X | X | 7 |
| Ryan C. E. | 2015 | X | X | X | X |  |  | X | X | X | 7 |
| Souche R. | 2018 | X | X | X | X |  |  | X | X | X | 7 |
| Vicente E. | 2020 | X | X | X | X |  |  | X | X | X | 7 |
| Waters J. A. | 2010 | X | X | X | X |  |  | X | X | X | 7 |
| Xourafas D. | 2017 | X | X | X | X |  |  | X | X | X | 7 |
| Yang S. J. | 2020 | X | X | X | X |  |  | X | X | X | 7 |
| Zhang J. | 2017 | X | X | X | X |  |  | X | X | X | 7 |
